# The dual PI3K/mTOR inhibitor BEZ235 restricts the growth of lung cancer tumors regardless of EGFR status, as a potent accompanist in combined therapeutic regimens

**DOI:** 10.1186/s13046-019-1282-0

**Published:** 2019-07-01

**Authors:** Yi-Ying Wu, Hung-Chang Wu, Jia-En Wu, Kuo-Yen Huang, Shuenn-Chen Yang, Si-Xuan Chen, Chao-Jung Tsao, Keng-Fu Hsu, Yuh-Ling Chen, Tse-Ming Hong

**Affiliations:** 1 Institute of Clinical Medicine, National Cheng Kung University, No.1, University Road, Tainan, 70101 Taiwan; 2 Clinical Medicine Research Center, National Cheng Kung University Hospital, National Cheng Kung University, Tainan, 70101 Taiwan; 3 Institute of Basic Medical Sciences, National Cheng Kung University, Tainan, 70101 Taiwan; 4 Department of Obstetrics and Gynecology, National Cheng Kung University Hospital, National Cheng Kung University, Tainan, 70101 Taiwan; 5 Institute of Oral Medicine, College of Medicine, National Cheng Kung University, Tainan, 70101 Taiwan; 6 Division of Hematology and Oncology, Department of Internal Medicine, Chi-Mei Medical Center, Yong Kang, Tainan, 71004 Taiwan; 7 Institute of Biomedical Sciences, Academia Sinica, Taipei, 11529 Taiwan; 8 Department of Hematology and Oncology, Chi-Mei Medical Center, Liouying, Tainan, 73657 Taiwan

**Keywords:** Lung cancer, BEZ235, PI3K/mTOR, EGFR, Combined therapy

## Abstract

**Background:**

Lung cancer is the most common cause of cancer-related mortality worldwide despite diagnostic improvements and the development of targeted therapies, notably including epidermal growth factor receptor (EGFR) tyrosine kinase inhibitors (TKIs). The phosphoinositide 3-kinase (PI3K)/AKT/mechanistic target of rapamycin (mTOR) signaling has been shown to contribute to tumorigenesis, tumor progression, and resistance to therapy in most human cancer types, including lung cancer. Here, we explored the therapeutic effects of co-inhibition of PI3K and mTOR in non-small-cell lung cancer (NSCLC) cells with different EGFR status.

**Methods:**

The antiproliferative activity of a dual PI3K/mTOR inhibitor BEZ235 was examined by the WST-1 assay and the soft agar colony-formation assay in 2 normal cell lines and 12 NSCLC cell lines: 6 expressing wild-type EGFR and 6 expressing EGFR with activating mutations, including exon 19 deletions, and L858R and T790 M point mutations. The combination indexes of BEZ235 with cisplatin or an EGFR-TKI, BIBW2992 (afatinib), were calculated. The mechanisms triggered by BEZ235 were explored by western blotting analysis. The anti-tumor effect of BEZ235 alone or combined with cisplatin or BIBW2992 were also studied in vivo.

**Results:**

BEZ235 suppressed tumor growth in vitro and in vivo by inducing cell-cycle arrest at G1 phase, but without causing cell death. It also reduced the expression of cyclin D1/D3 by regulating both its transcription and protein stability. Moreover, BEZ235 synergistically enhanced cisplatin-induced apoptosis in NSCLC cells by enhancing or prolonging DNA damage and BIBW2992-induced apoptosis in EGFR-TKI–resistant NSCLC cells containing a second TKI-resistant EGFR mutant.

**Conclusions:**

The dual PI3K/mTOR inhibition by BEZ235 is an effective antitumor strategy for enhancing the efficacy of chemotherapy or targeted therapy, even as a monotherapy, to restrict tumor growth in lung cancer treatment.

**Electronic supplementary material:**

The online version of this article (10.1186/s13046-019-1282-0) contains supplementary material, which is available to authorized users.

## Background

Lung cancer remains the leading cause of cancer-related death worldwide [1]. It is considered a highly heterogeneous, aggressive, and relentlessly progressive disease with few treatment options and poor survival, largely because most (~ 70%) cases are diagnosed at an advanced stage. Although surgery for early-stage patients offers the best prognosis, patients with advanced disease treated with chemotherapies, including platinum- and Taxol-based treatments, usually develop drug resistance or cancer progression. The five-year survival of patients with lung cancer is currently less than 18% [2, 3].

Over the last 10 years, there have been concerted research efforts to develop molecular histological techniques and targeted therapies for cancer. Small-molecule epidermal growth factor receptor tyrosine kinase inhibitors (EGFR-TKIs) that compete with ATP binding and inhibit downstream signal transduction have been developed as part of these efforts. These EGFR-TKIs, such as gefitinib and erlotinib, have proven to be effective in non-small cell lung cancer (NSCLC) patients carrying specific activating mutations in the tyrosine kinase domain of EGFR, mostly within exon 18–21, such as the L858R point mutation and deletions in exon 19 [4–6]. Unfortunately, not all EGFR kinase mutations are associated with hypersensitivity to the regimen, and almost all patients develop drug resistance after long-term EGFR-TKI administration [7]. Recent studies have revealed several molecular mechanisms underlying EGFR-TKI resistance, including a second mutation at T790 M in exon 20 of EGFR, activation of alternative receptor tyrosine kinases (RTKs) or PI3K signaling, small-cell lung cancer (SCLC) transformation, and epithelial-mesenchymal transition (EMT) [8–10].

Dysregulation of the phosphoinositide 3-kinase (PI3K)/AKT/mechanistic target of rapamycin (mTOR) axis has been implicated in most human cancer types and shown to contribute to tumorigenesis, tumor progression, and resistance to therapy [11–13]. PI3K signaling, induced by activation of RTKs and G-protein-coupled receptors (GPCRs), regulates many cellular functions, including cell proliferation, survival, metabolism, motility, and angiogenesis. Accumulating evidence suggests that PI3K/Akt/mTOR signaling is involved in the resistance to many cancer therapies, including chemotherapy, RTK inhibitors, anti-angiogenic therapy, mitogen-activated protein kinase (MAPK) pathway inhibitors, hedgehog pathway inhibitors, and immunotherapy [14]. The major mechanisms underlying EGFR-TKI resistance—the EGFR T790 M mutation, activation of alternative RTKs (e.g., HER2/Neu, ERBB3, MET, AXL, IGF-1R, PIK3CA) and loss of PTEN (phosphatase and tensin homolog)—also ultimately activate PI3K/Akt/mTOR signaling [8]. Therefore, inhibition of the PI3K/Akt/mTOR pathway is critical for cancer therapy.

BEZ235 (dactolisib), a synthetic imidazoquinoline derivative, is an orally bioavailable, dual PI3K/mTOR inhibitor that selectively inhibits class I PI3K (p110α, −β, −δ and -γ), mTORC1 and mTORC2 by reversibly binding to the ATP-binding sites of kinases and inhibiting their catalytic activity and signaling [15]. BEZ235 has been reported to exert excellent antitumor effects against many cancers [16–19] and is currently undergoing Phase I/II clinical trials for the treatment of solid tumors. Here, we investigated the antitumor effects of dual PI3K/mTOR inhibition by BEZ235 as a monotherapy or in combination with cisplatin or BIBW2992 (afatinib), a second generation EGFR-TKI, in NSCLC cells with different EGFR status or sensitivity to EGFR-TKIs.

## Methods

### Cell lines and reagents

A panel of human NSCLC cell lines, CL83, CL141, CL152, CL25 and CL97, was established in our laboratory from pleural effusion samples from lung cancer patients; their clinical characteristic, gene mutations, and responses to EGFR-TKIs were described previously [20]. A549, H3255, and H1975 human NSCLC cell lines and human bronchial fibroblasts (HBFs) were purchased from the American Type Culture Collection (ATCC; Manassas, VA USA). Human normal bronchial epithelial (NBE) cells were from Dr. Pan-Chyr Yang (Institute of Biomedical Sciences, Academia Sinica, Taipei, Taiwan). The primary NSCLC cell lines, ZSY and LIJ, were kind gifts from Dr. Wu-Chou Su (Institute of Molecular Medicine, National Cheng Kung University, Taiwan). PC-9 and gefitinib-resistant PC-9 cells (PC-9 IR) [21] were obtained from Dr. James Chih-Hsin Yang (Institute of Oncology, National Taiwan University, Taiwan). PC9, PC9-IR and CL25 cells harbor an activating EGFR mutation (delE746_A750), whereas H3255 cells express the EGFR L858R point mutation. H1975 and CL97 cells have the second TKI-resistance mutation, EGFR-T790 M. Cells were cultured in RPMI-1640 media (Invitrogen, Eugene, OR, USA) supplemented with 10% fetal bovine serum at 37 °C in a humidified 5% CO_2_ atmosphere. BEZ235, BIBW2992 (afatinib), and cisplatin were purchased from Selleck Chemicals (Houston, TX, USA).

### WST-1 assays

Cells (3 × 10^3^/well) were cultured in 96-well plates and allowed to adhere overnight. For cell proliferation analysis, cell numbers were determined at 0, 1, 2, 3, and 4 days by measuring the metabolic conversion of the water-soluble tetrazolium salt, WST-1 (Roche Diagnostics, Basel, Switzerland). WST-1 was added to each well, after which the cells were further incubated for 1 h at 37 °C before measurement of optical density at 450 nm using an ELISA reader. For growth-inhibition analysis, cells were incubated for 48 or 72 h in the presence of different concentrations of BEZ235 and cisplatin or BIBW2992, alone or together. Cell viability was also assessed using the WST-1 assay. Drug concentrations resulting in 50% growth inhibition (IC_50_) and combination index (CI) values were calculated using CalcuSyn software (Biosoft, Cambridge, United Kingdom). CI values < 1, = 1, and > 1 indicate synergistic, additive, and antagonistic effects, respectively.

### Soft agar colony-formation assay

NSCLC cells (500 cells/well) in media containing 0.35% agarose were seeded in triplicate onto six-well plates coated with 0.7% agarose. Anchorage-independent growth was assessed after incubation for 14 days in complete media, with or without 100 nM BEZ235, replaced every 3 days. At the end of the incubation period, colonies were stained with 0.01% crystal violet in 70% ethanol. After carefully removing the crystal violet and rinsing with water, colonies were photographed and counted under an inverted microscope.

### Western blot analysis

Cells were lysed on ice for 30 min in RIPA buffer (0.5% sodium deoxycholate, 0.1% sodium dodecyl sulfate [SDS], 1% Triton X-100 in 1x Tris-buffered saline [TBS]) containing 100 μM Na_3_VO_4_, 50 mM NaF, 30 mM Na pyrophosphate, and a 25-fold dilution of a stock solution, prepared from one mini protease inhibitor cocktail tablet (Roche Diagnostics, Basel, Switzerland) dissolved in 2 ml of distilled water. Proteins in whole-cell lysates were separated by SDS-PAGE (polyacrylamide gel electrophoresis), transferred to polyvinylidene membranes (Millipore, Billerica, MA, USA), and probed with primary antibodies. The following primary antibodies were used: anti-β-actin monoclonal antibody (Sigma, St. Louis, MO, USA); anti-phospho-Akt (Ser473), Akt, phospho-Erk1/2, Erk1/2, phospho-Stat3 (Y705), Stat3, phospho-p70S6K (Thr389), p70S6K, phospho-4EBP1 (Ser65), phospho-GSK3β (Ser9), phospho-GSK3β (Tyr216), GSK3β, cleaved PARP, caspase 3, cyclin D1, cyclin D3 and γ-H2A.X (Cell Signaling Technology, Beverly, MA, USA); anti-4EBP1 polyclonal antibodies (GeneTex, San Antonio, TX, USA); and anti-LC3 polyclonal antibody (Abgent, San Diego, CA, USA). Antibodies were diluted in TBS (pH 7.5) containing 0.05% (v/v) Tween-20 and 5% non-fat milk. Blots were incubated with the appropriate horseradish peroxidase-conjugated secondary antibodies (GE Healthcare Life Sciences, Piscataway, NJ, USA), and immunoreactive proteins were visualized using enhanced chemiluminescence (ECL) staining. The blot density was analyzed by ImageJ software (NIH, Bethesda, MD, USA) and then the expression level of indicated protein was normalized to the internal control, β-actin. The relative expression level to the control of each experiment was shown below the indicated blots.

### Cell-cycle analysis

Following BEZ235 treatment, cells were fixed in 70% ethanol, stained with 10 μg/ml of propidium iodide containing 20 μg/ml RNase A, and then analyzed by flow cytometry using a FACSCalibur system (Becton Dickinson, San Jose, CA, USA). For each analysis, 10,000 cells were counted, and the percentage of cells in each phase was calculated using ModFit LT software (Verity Software House, Inc., Topsham, ME, USA).

### Reverse transcription-polymerase chain reaction (RT-PCR)

Total RNA was prepared using the TRIzol reagent (Invitrogen). One microgram of isolated total RNA was reverse transcribed for 60 min at 50 °C using Super Script III Reverse Transcriptase (Invitrogen) and random hexamer primers (Applied Biosystems, Foster City, CA, USA) in the presence of an RNase inhibitor (Invitrogen). Expression of *CCND1, CCND3* and *GAPDH* mRNA in BEZ235-treated cells was measured by SYBR green-based real-time quantitative PCR using Fast SYBR Green Master Mix and the Applied Biosystems StepOne Real-Time PCR System (Applied Biosystems). Reaction mixes (10 μl total volume) contained 1 μl cDNA (diluted 1:10), 0.2 μM forward primer, 0.2 μM reverse primer, and 1x Fast SYBR Green Master Mix. Thermocycling conditions were as follows: pre-incubation at 95 °C for 2 min, followed by 40 cycles of denaturation at 95 °C for 3 s and annealing/extension at 60 °C for 30 s. *CCND1/CCND3* mRNA levels relative to those of GAPDH were defined as -∆CT = −[CT_CCND1/3_ – CT_GAPDH_], and the CCND1 or CCND3 cDNA/GAPDH cDNA ratio was calculated as 2^-∆CT^. Relative expression of CCND1 or CCND3 mRNA is presented as the expression in BEZ235-treated cells relative to that in vehicle (DMSO)-treated control cells. No-template controls were included in each assay.

### Tumor xenograft model

The tumor model was established by subcutaneously inoculating 6-week-old male Balb/c nude mice (NARLabs, Taipei, Taiwan) in the right flank with 2 × 10^6^ H1975 cells in a total volume of 0.1 ml sterile phosphate-buffered saline (PBS; pH 7.4) on day 0. After tumors had reached ~ 50 mm^3^, mice were randomized into the following two groups (*n* = 8/group): 1) 25 mg/kg BEZ235 and 2) vehicle control (0.5% methylcellulose, 0.1% Tween-80 in sterile water containing the same percentage of DMSO). BEZ235 diluted in the buffer with 0.5% methylcellulose and 0.1% Tween-80 retains most of its drug enzymatic activity (Additional file 1: Figure S1a). Mice were treated daily with the indicated regimen, administered by oral gavage, for 18 days (200 μl/mouse). For combined treatment with BEZ235 and BIBW2992 [22], mice were randomized into the following four groups (*n* = 5/group): 1) vehicle control, 2) 25 mg/kg BEZ235 only, 3) 5 mg/kg BIBW2992 only, 4) 25 mg/kg BEZ235 and 5 mg/kg BIBW2992. Mice were treated with the indicated regimen for 19 days (200 μl/mouse). For combined treatment with BEZ235 and cisplatin (Additional file 1: Figure S1b and c), mice were subcutaneously inoculated in the right flank with 2 × 10^6^ CL141 cells in a total volume of 0.1 ml sterile PBS (pH 7.4), and then treated with BEZ235 or cisplatin after tumors had reached ~ 50 mm^3^. For these experiments, mice were randomized into the following four groups (n = 5/group): 1) vehicle control, 2) 25 mg/kg BEZ235, 3) 2.5 mg/kg cisplatin, 4) 25 mg/kg BEZ235 and 2.5 mg/kg cisplatin. Mice were treated with the indicated regimen for 19 days (200 μl/mouse). BEZ235 (BEZ235 only and BEZ235 plus cisplatin groups) or vehicle buffer (control and cisplatin only groups) was administered daily by oral gavage, whereas cisplatin (cisplatin only and BEZ235 plus cisplatin groups) or PBS (control and BEZ235 only group) was administered twice weekly by intraperitoneal injection. Tumor size was measured twice a week using calipers, and tumor volume was calculated according to the formula, V = ab^2^/2, where a is the length and b is the width of the tumor. Body weight was also measured twice a week. At the end of the study, mice were sacrificed by CO_2_ inhalation. All animal experiments were performed according to guidelines of the Animal Care Ethics Commission using a standard protocol approved by the Laboratory Animal Center, Institute of Biomedical Sciences, Academia Sinica, Taipei, Taiwan and the Laboratory Animal Center, College of Medicine, National Cheng Kung University, Tainan, Taiwan.

### Statistical analysis

Data are presented as means ± standard deviation (SD) or standard errors of the mean (SEM), as appropriate. The significance of differences between two groups was determined by Student’s t-test using GraphPad Prism software (Version 5.0). All statistical tests were two-sided, and *P*-values < 0.05 were considered statistically significant.

## Results

### BEZ235 exerts potent in vitro antigrowth effects against NSCLC cell lines with different EGFR status

**Fig. 1:**
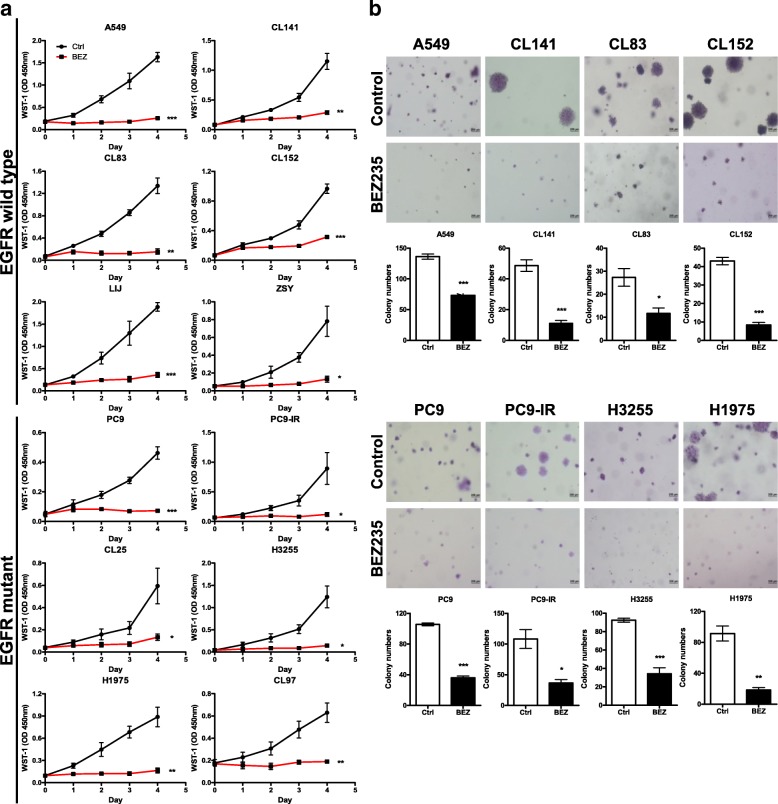
BEZ235 suppresses growth of NSCLC cells. **a** BEZ235 blocks proliferation of NSCLC cell lines expressing either wild-type EGFR or activating mutants of EGFR. Proliferation of NSCLC cells treated with vehicle control (ctrl) or 100 nM BEZ235 (BEZ) was assessed using the WST-1 assay. A549, CL141, CL83, ZSY, LIJ and CL152 cell lines expressed wild-type EGFR, whereas PC9, PC9-IR, CL25, H3255, H1975 and CL97 cell lines expressed activating mutants of EGFR. Of these, PC9-IR, H1975 and CL97 were resistant to gefitinib, and CL25 was partially sensitive to it. Three independent experiments were performed in triplicate. Values are reported as means ± SD (*, *P* < 0.05; **, *P* < 0.01; ***, *P* < 0.001; Student’s t-test). **b** BEZ235 suppresses the anchorage-independent growth of both EGFR-wild type and EGFR-mutant NSCLC cells. Cells were seeded at 500 cells/plate and grown in soft agar in medium containing vehicle (DMSO) or 100 nM BEZ235 for 14 days, after which colonies were photographed and counted. Three independent experiments were performed in triplicate. Values are reported as means ± SD (*, *P* < 0.05; **, *P* < 0.01; ***, *P* < 0.001; Student’s t-test)

Based on the premise of aberrant EGFR and PI3K/mTOR signaling in lung cancer [4, 6], we investigated the antiproliferative activity of the dual PI3K/mTOR inhibitor BEZ235 in 12 NSCLC cell lines: 6 expressing wild-type EGFR and 6 expressing EGFR with activating mutations, including exon 19 deletions, and L858R and T790 M point mutations. As shown in Table 1 and Additional file 1: Figure S2, BEZ235 exerted potent growth-inhibitory activity against all tested NSCLC cell lines, with 50% inhibitory concentration (IC_50_) values ranging from 6.86 to 193.40 nM. By comparison, IC_50_ values for normal human fibroblast and normal human bronchial epithelia were greater than 10 μM. Notably, both CL141 and CL152 cell lines, which have lost PTEN expression, remained sensitive to BEZ235 treatment. In addition, BEZ235 also effectively suppressed the growth of PC9-IR, H1975 and CL97 cell lines, with acquired EGFR-TKI resistance. The parental PC9 cell line and its variant, PC9-IR, which is gefitinib-resistant by virtue of having gained EMT ability [21], exhibited similar sensitivity to BEZ235. Moreover, treatment with 100 nM BEZ235 almost completely suppressed the growth of all tested NSCLC cell lines for at least 4 days (Fig. 1a). However, BEZ235 did not decrease the number of NSCLC cells, which returned to proliferating normally upon removing BEZ235 after treatment for 3 days (Additional file 1: Figure S3). BEZ235 also significantly suppressed anchorage-independent cell growth in NSCLC cells (Fig. 1b).

**Table 1:** IC_50_ of BEZ235 for growth inhibition in human lung cancer cells

Cell line	Histology	EGFR status	PTEN status	KRAS status	Response to gefitinib	IC_50_ for BEZ235 (nM)
HBF^a^	Normal	WT	Normal	WT	Resistance	> 10 μM
NBE^b^	Normal	WT	Normal	WT	Resistance	> 10 μM
A549	Adenocarcinoma	WT	Normal	G12S	Resistance	31.57 ± 12.76
CL141	Adenocarcinoma	WT	Loss	WT	Resistance	43.30 ± 7.27
CL83	Adenocarcinoma	WT	Normal	WT	Resistance	19.94 ± 5.46
ZSY	Adenocarcinoma	WT	ND	ND	Resistance	6.86 ± 3.26
LIJ	Adenocarcinoma	WT	ND	ND	Resistance	193.40 ± 52.76
CL152	Squamous cell carcinoma	WT	Loss	WT	Resistance	45.49 ± 15.36
PC9	Adenocarcinoma	Exon 19 deletion	Normal	WT	Sensitive	19.81 ± 3.44
PC9IR	Adenocarcinoma	Exon 19 deletion	Normal	WT	Resistance	28.75 ± 0.98
CL25	Adenocarcinoma	Exon 19 deletion	Normal	WT	Partial sensitive	56.20 ± 5.66
H3255	Adenocarcinoma	L858R	Normal	WT	Sensitive	12.60 ± 2.53
H1975	Adenocarcinoma	L858R/T790 M	Normal	WT	Resistance	16.24 ± 3.63
CL97	Adenocarcinoma	G719A/T790 M	Normal	WT	Resistance	40.89 ± 27.42

^a^Human bronchial fibroblast

^b^Human normal bronchial epithelia

ND, not determined

### BEZ235 blocks PI3K/mTOR signaling and induces G0/G1 growth arrest by decreasing cyclin D1/D3 in NSCLC cells

**Fig. 2:**
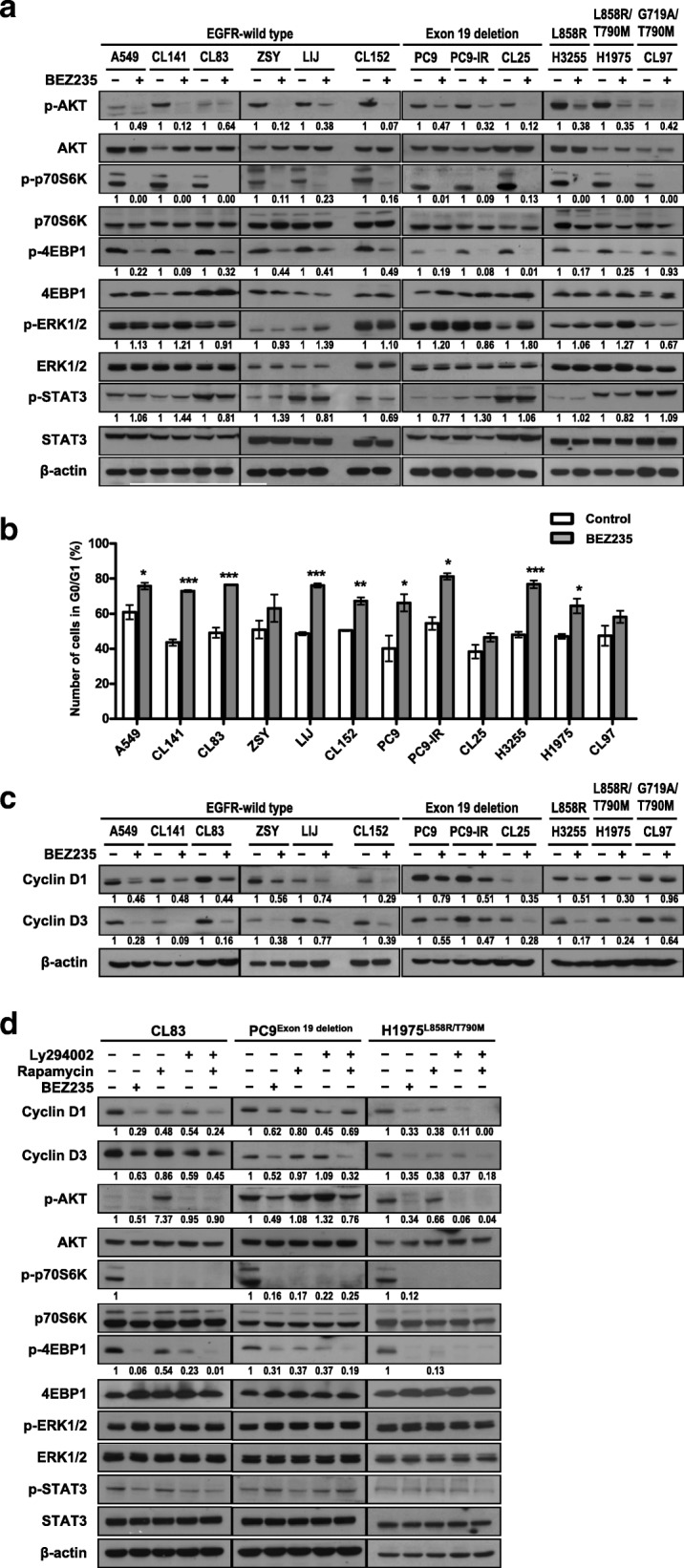
The dual PI3K/mTOR inhibitor BEZ235 causes cell arrest at G0/G1 phase by decreasing cyclin D. **a** BEZ235 inhibits both PI3K and mTOR signaling in NSCLC cell lines expressing either wild-type EGFR or activating mutants of EGFR. NSCLC cells were treated with 100 nM BEZ235 for 6 h, and the levels of phosphorylated AKT, p70S6K, 4EBP1, ERK1/2, and STAT3 were examined. β-actin was used as an internal control. **b** BEZ235 causes cell arrest at G0/G1 phase. Cells were treated with 333 nM BEZ235 for 24 h, and then the cell-cycle distribution was analyzed by flow cytometry. The percentage of cells in the G0/G1 phase of the cell cycle was determined. Values are the results of three independent experiments and are reported as means ± SD (*, *P* < 0.05; **, *P* < 0.01; ***, *P* < 0.001; Student’s t-test). **c** BEZ235 reduces expression of cyclin D1 and cyclin D3. Cells were treated as described in a. **d** Dual blockade of PI3K/mTOR pathways strongly diminishes cyclin D1 and cyclin D3 levels. Cells were treated for 6 h with 20 μM LY294002, rapamycin, or both, and molecular signaling was compared with that in cells treated with 100 nM BEZ235. Cell lysates were analyzed by Western blotting using the indicated antibodies. The related expression levels of protein, quantified by ImageJ as described in Materials and Methods, were shown below their corresponding blots

To further validate the effects of BEZ235 on EGFR and PI3K/mTOR signaling pathways, we treated all NSCLC cell lines with 100 nM BEZ235 for 6 h. As shown in Fig. 2a, phosphorylated levels of the PI3K downstream target, AKT, and the mTOR signaling effectors, p70S6K (ribosomal protein S6 kinase) and 4EBP1 (eukaryotic translation initiation factor 4E binding protein 1), were reduced in all drug-treated cell lines, whereas the levels of phosphorylated ERK1/2 (extracellular signal-regulated kinase 1/2) and STAT3 (signal transducer and activator of transcription 3) were unaffected. Given the striking antigrowth effects of BEZ235, we next analyzed apoptotic and autophagic cell death processes, and the cell-cycle distribution in BEZ235-treated NSCLC cells. Neither levels of the apoptotic markers, cleaved poly (ADP-ribose) polymerase (PARP) and active caspase 3, nor the level of the autophagic marker, LC3-II, were changed in NSCLC cells after a 24-h treatment with BEZ235; the only exception was an increase in LC3-II in A549 cells (Additional file 1: Figure S4a). Consistent with these results, a flow cytometry analysis of cell-cycle progression showed that the percentage of cells in the sub-G1 phase was not significantly altered after 24-h treatment with 333 nM BEZ235 (Additional file 1: Figure S4b). Notably, BEZ235 caused accumulation of cells in the G0/G1 phase (Fig. 2b). We further found a reduction in the expression of cyclin D1 and cyclin D3 in all BEZ235-treated NSCLC cells after 6 h of treatment (Fig. 2c). Cyclin D2 was undetectable in these NSCLC cells (data not shown). To dissect the role of PI3K and mTOR signaling in the regulation of cyclin D members, we further compared the inhibitory functions of BEZ235 with those of LY294002 and rapamycin, specific inhibitors of PI3K and mTOR, respectively (Fig. 2d). These experiments showed that, in NSCLC cells with varying EGFR status, BEZ235 or the combination of LY294002 and rapamycin efficiently reduced the expression of cyclin D1 and cyclin D3. LY294002 or rapamycin alone mild decreased the expression of these cyclins in CL83 whereas only cyclin D1 was decreased by LY294002 and slightly reduced by rapamycin in PC9. In H1975, both of LY294002 and rapamycin could efficiently reduce the levels of cyclin D1 and cyclin D3. Besides, combined with LY294002 and rapamycin inhibited the phosphorylation of AKT, p70S6K and 4EBP1, a signal-blocking profile similar to that of BEZ235, in NSCLC cells. Under the LY294002 treatment, CL83 and H1975 had low levels of phospho-AKT, phospho-p70S6K and phospho-4EBP1 while PC9 had low levels of phospho-p70S6K and phospho-4EBP1 but increased phosphorylated of AKT. Moreover, treatment with rapamycin alone attenuated the phosphorylation of p70S6K and 4EBP1, but not that of AKT in NSCLC cells, and even induced the phosphorylation of AKT in CL83. It is known that mTOR inhibition may induce feedback activation of Akt signaling [12]. Although blocking PI3K or mTOR alone by LY294002 and rapamycin, respectively, may efficiently suppress the expression of cyclin Ds and the phosphorylation of Akt, p70S6k and 4EBP1 in certain NSCLC cells, the suppressive effect by co-blockage of PI3K and mTOR was more comprehensive in NSCLC. Taken together, these results confirm dual PI3K/mTOR pathway blockade by BEZ235, as evidenced by diminished cyclin D1 and cyclin D3, resulting in G0/G1 growth arrest in NSCLC cells, regardless of EGFR status.

### BEZ235 leads to both proteosomal and transcriptional downregulation of cyclin D1 and D3

**Fig. 3:**
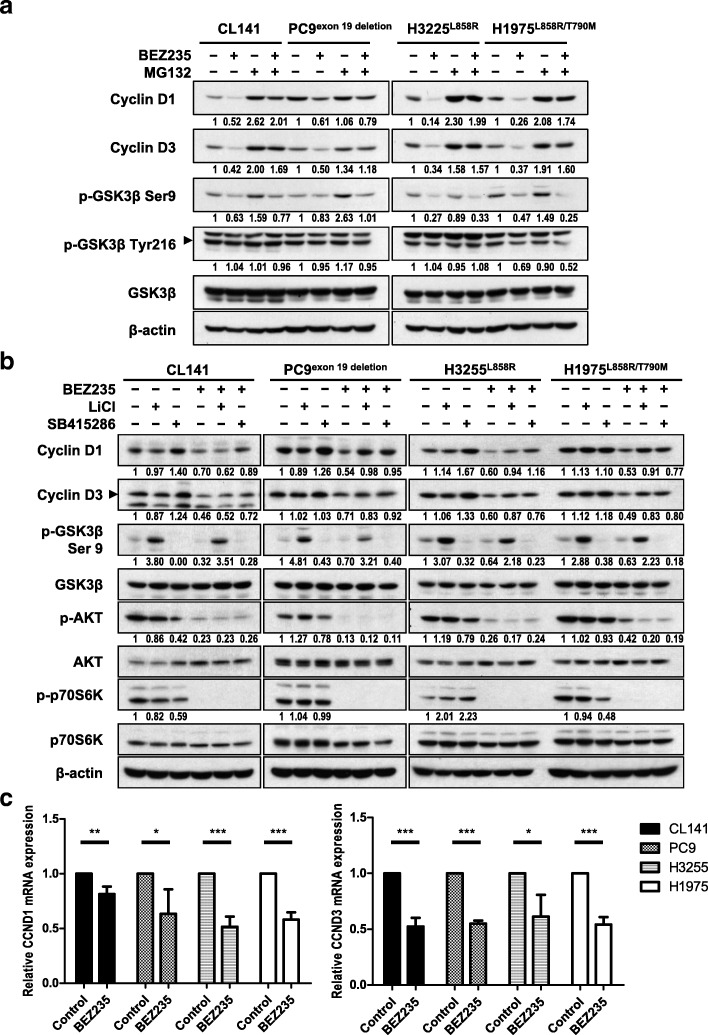
BEZ235 causes proteosomal degradation and transcriptional downregulation of cyclin D1 and cyclin D3. **a** BEZ235 induces the proteosome-mediated degradation of cyclin D. NSCLC cells expressing either wild-type EGFR or activating mutants of EGFR were co-treated with 20 μM MG132 and 100 nM BEZ235 for 6 h. The levels of cyclin D1/D3 and phosphorylated GSK3β at Ser9 or Tyr216 were determined by Western blotting using the indicated antibodies. **b** GSK3β inhibitors partially reverse the BEZ235-induced downregulation of cyclin D1 and D3. NSCLC cells were treated with 100 nM BEZ235 and 20 mM LiCl or 20 μM SB415286 for 6 h. Cell lysates were analyzed by Western blotting using the indicated antibodies. The related expression levels of protein, quantified by ImageJ as described in Materials and Methods, were shown below their corresponding blots. **c** BEZ235 reduces mRNA expression of cyclin D1 and D3 in NSCLC cells. *CCND1* and *CCND3* mRNA expression levels in cells treated with BEZ235 cells for 6 h compared with those in DMSO-treated control cells were determined by qRT-PCR and normalized to mRNA levels of GAPDH, used as an internal control. Data are shown as means ± SD (*, *P* < 0.05; **, *P* < 0.01; ***, *P* < 0.001; Student’s t-test)

The decrease in cyclin D induced by BEZ235 could reflect protein degradation or transcriptional downregulation. Consistent with a role for protein degradation in this effect of BEZ235, co-treatment of NSCLC cells with the proteosome inhibitor MG132 partially rescued BEZ235-induced downregulation of cyclin D1 and cyclin D3 (Fig. 3a). It is known that cyclin D1 is phosphorylated at Thr286 by GSK3β, resulting in its proteasome-mediated protein degradation [23], and further that AKT phosphorylates GSK3β at Ser9, inhibiting its activity [24]. BEZ235 also suppressed the Ser9 phosphorylation of GSK3β by inhibiting PI3K/AKT signaling. The Tyr216 phosphorylation of GSK3β, facilitating the kinase activity of GSK3β, had no change in CL141, PC9 and H3255 and even also decreased in H1975 upon BEZ235 treatment. Co-treatment of NSCLC cells with LiCl or SB415286, which block the activity of GSK3β, partially restored cyclin D1 and cyclin D3 expression in BEZ235-treated NSCLC cells as well, except cyclin D1 in CL141 co-treated with LiCl (Fig. 3b). LiCl, inhibited the GSK3β activity by unknown mechanism, promoted the Ser9 phosphorylation of GSK3β while SB415286, a selective, ATP-competitive GSK3 inhibitor, did not. Besides protein degradation, we further found that BEZ235 treatment decreased the mRNA levels of both cyclin D1 (*CCND1*) and cyclin D3 (*CCND3*) in NSCLC cells (Fig. 3c). However, β-catenin, a well-known transcriptional activator of cyclin D1 that is phosphorylated by GSK3β and then degraded by the proteasome, was not affected by BEZ235 in NSCLC cells (Additional file 1: Figure S5). Collectively, these observations indicate that BEZ235 causes the decrease of cyclin D1 and cyclin D3 by both of β-catenin-independent transcriptional downregulation and GSK3β-mediated proteosomal degradation.

### In vivo antitumor effect of BEZ235 in xenograft mice

**Fig. 4:**
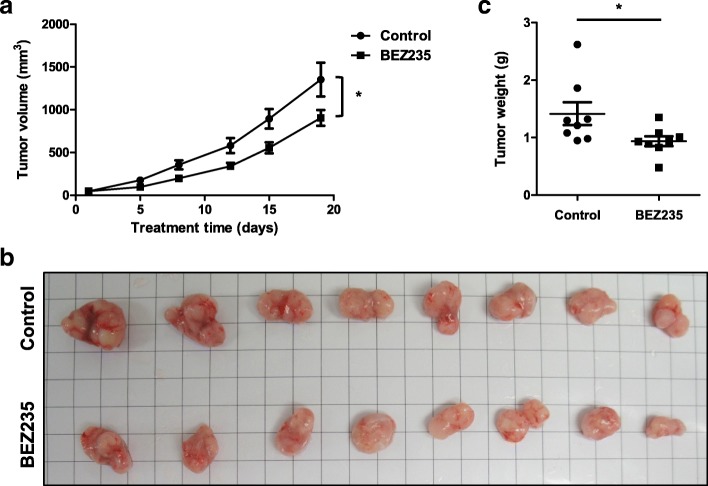
BEZ235 exerts antitumor effects in vivo in an NSCLC mouse xenograft model. **a** Mice with a subcutaneous H1975 cell xenograft tumor were orally administered 25 mg/kg BEZ235 daily. Volumes of tumors from vehicle control (●) and BEZ235 (■) groups (*n* = 8/group) were measured twice a week. Data are presented as means ± SEM (error bars; *, P < 0.05; Student’s t-test). Images of tumors (**b**) and weights of tumors (**c**) from mice with an H1975 cell xenograft after treatment with vehicle control or BEZ235 for 18 days. Data are presented as means ± SEM (error bars; *, *P* < 0.05; Student’s t-test)

Based on the potent antigrowth effects of BEZ235 in vitro, we next examined the in vivo antitumor efficacy of BEZ235 in xenograft mice. To this end, H1975 cells were subcutaneously inoculated into the right flank of nude mice, which were then treated daily with 25 mg/kg of BEZ235 or vehicle control by oral gavage after tumors had reached ~ 50 mm^3^. These experiments showed that BEZ235 treatment significantly reduced both tumor size and tumor weight (Fig. 4).

### Combined treatment with BEZ235 and cisplatin is an effective antitumor strategy

**Fig. 5:**
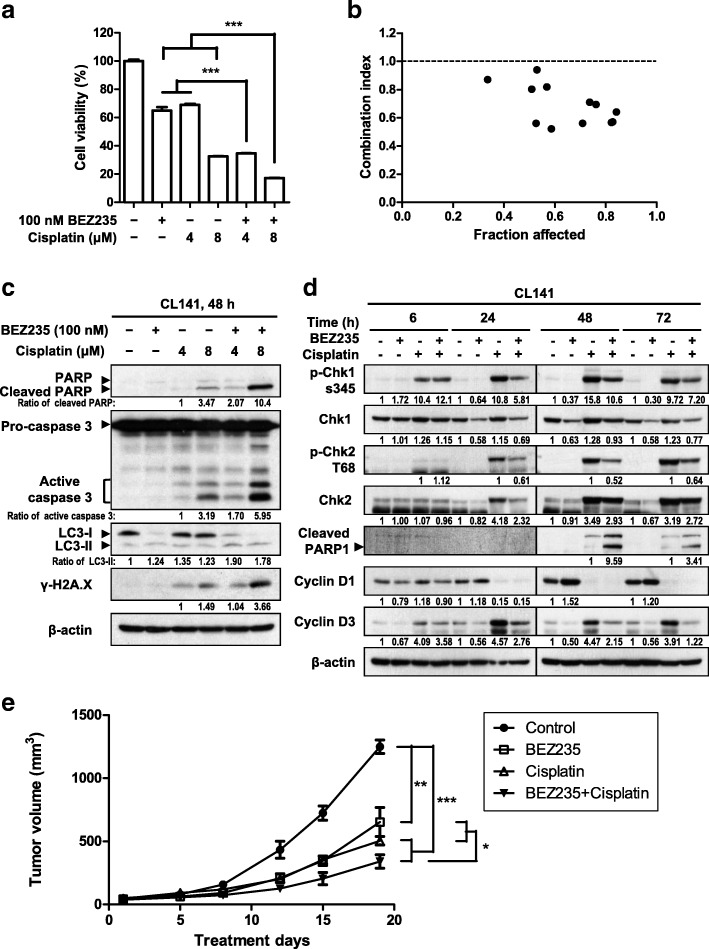
BEZ235 synergistically potentiates cell death induced by cisplatin in NSCLC cells. **a** CL141 cells were treated with the indicated concentrations of cisplatin and BEZ235, either alone or together, for 48 h. Viability was analyzed using the WST-1 assay. **b** Combined treatment with cisplatin and BEZ235 as in Fig. S6a exerts synergistic effects. The combination index was calculated as described in Materials and Methods. **c** BEZ235 potentiates cisplatin-induced apoptosis and DNA damage. CL141 cells were treated with cisplatin and BEZ235 at the indicated concentrations for 48 h. Levels of the apoptotic indicators, cleaved PARP and active caspase 3, the autophagic marker LC3-II, and γ-H2A.X in cell lysates were analyzed by Western blotting. **d** BEZ235 suppresses Chk1 and Chk2 activation. CL141 cells were treated with 8 μM cisplatin and 100 nM BEZ235, either alone or together, for the indicated time. Levels of phosphorylated Chk1, Chk1, phosphorylated Chk2, Chk2, cleaved PARP1, cyclin D1, cyclin D3, and β-actin in cell lysates were analyzed by Western blotting using the indicated antibodies. The related expression levels of protein, quantified by ImageJ as described in Materials and Methods, were shown below their corresponding blots. **e** The combination of BEZ235 and cisplatin exerts dramatic antitumor effects in NSCLC xenograft mice in vivo. BEZ235 (25 mg/kg) was orally administered daily, and cisplatin (2.5 mg/kg) was intraperitoneally injected twice per week in mice bearing a subcutaneous CL141 cell xenograft tumor. Volumes of tumors from vehicle control (●), BEZ235 (□), cisplatin (△) and BEZ235 + cisplatin (▼) groups (*n* = 5/group) were measured twice a week. Data are presented as means ± SEM (error bars; *, *P* < 0.05; **, *P* < 0.01; ***, *P* < 0.001; Student’s t-test)

Because the PI3K/Akt/mTOR pathway acts as an important survival signal [25], blockade of PI3K/Akt/mTOR signaling may enhance the efficiency of traditional chemotherapy. As shown in Fig. 5a-b and Additional file 1: Figure S6a–e, BEZ235 synergistically enhanced cisplatin-induced cytotoxicity in CL141, CL83, and CL152 cells. It also increased the levels of the apoptotic indicators, cleaved PARP and caspase 3, and the autophagic marker, LC3-II, measured 48 h after cisplatin treatment, except the LC3-II level in CL152 (Fig. 5c and Additional file 1: Fig. S6f and g). Cisplatin-induced DNA damage, assessed by measuring γ-H2A.X levels, was also enhanced by BEZ235. Moreover, whereas cisplatin did not cause DNA damage or apoptosis after a 6-h treatment, γ-H2A.X levels in NSCLC cells were increased after 24 h of cisplatin treatment and were maintained for 48 h, subsequently resulting in apoptosis. BEZ235 did not increase γ-H2A.X levels 24 h post cisplatin treatment, but did dramatically increase the levels of γ-H2A.X and active caspase 3 after 48 h of cisplatin treatment (Additional file 1: Fig. S6 h). Because pathways mediated by the protein kinases Chk1 and Chk2 are responsible for triggering DNA damage checkpoints [26], we studied the effects of BEZ235 on the activation of Chk1 and Chk2. We found that BEZ235 reduced both phosphorylated and total levels of Chk1 and Chk2 24 h post treatment (Fig. 5d). This suggests that BEZ235 causes accumulation of DNA damage or promotes cisplatin-induced DNA damage by suppressing Chk1 and Chk2 pathways, and thereby enhances cell apoptosis. In addition, BEZ235 promoted cisplatin-induced cytotoxicity in H1975 and CL97 NSCLC cells expressing the EGFR-T790 M mutant as well (Additional file 1: Figure S6i).

We then examined the in vivo antitumor efficacy of combined treatment with BEZ235 and cisplatin in xenograft mice. Nude mice were subcutaneously inoculated in the right flank with CL141 cells and, after tumors reached ~ 50 mm^3^, were treated with vehicle control, 25 mg/kg of BEZ235, 2.5 mg/kg of cisplatin or both BEZ235 and cisplatin, as described in Materials in Methods. Treatment with BEZ235 or cisplatin significantly reduced tumor volume compared with the control group. Notably, co-treatment with BEZ235 and cisplatin exerted more effective antitumor effects than either agent alone (Fig. 5e). Taken together, our results suggest that BEZ235 sensitized NSCLC cells to the pro-apoptotic effects of cisplatin in association with an increase in DNA damage. These observations suggest that combined treatment with BEZ235 and cisplatin is an effective antitumor strategy, regardless of EGFR status.

### Combined EGFR-targeted therapy (BIBW2992) and BEZ235 is also an effective antitumor strategy for EGFR-TKI–resistant NSCLC cells expressing the EGFR T790 M point mutation

**Fig. 6:**
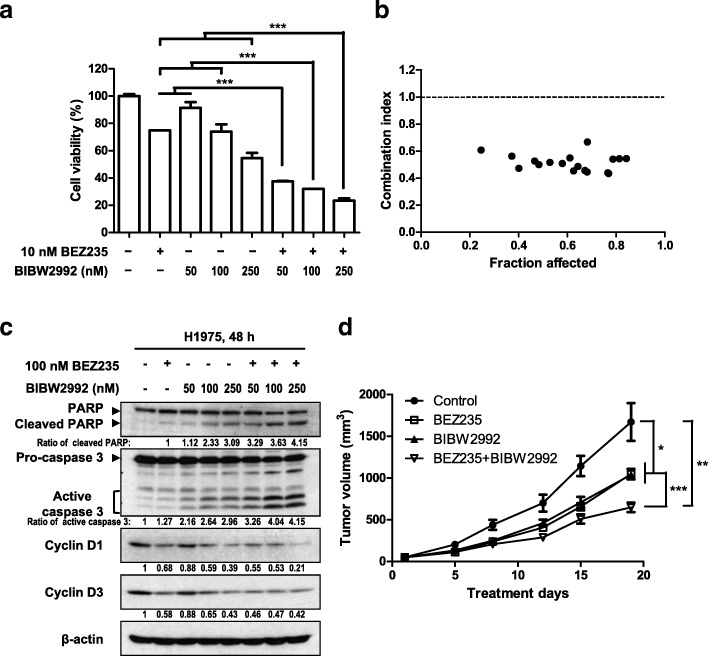
The combination of BEZ235 and BIBW2992 synergistically induces apoptosis in H1975 cells. **a** H1975 cells were treated with the indicated concentrations of BIBW2992 and BEZ235, either alone or together, for 72 h. Viability was analyzed using the WST-1 assay. **b** Combined treatment with BIBW2992 and BEZ235 as in Fig. S7a exerts synergistic effects. The combination index was calculated as described in Materials and Methods. **c** BEZ235 promotes BIBW2992-induced apoptosis. H1975 cells were treated with cisplatin and BEZ235 at the indicated concentrations for 48 h. Cell lysates were analyzed by Western blotting using the indicated antibodies. The related expression levels of protein, quantified by ImageJ as described in Materials and Methods, were shown below their corresponding blots. **d** The combination of BEZ235 and BIBW2992 exerts dramatic antitumor effects in NSCLC xenograft mice in vivo. BEZ235 (25 mg/kg) and BIBW2992 (5 mg/kg) were orally administered daily in mice bearing subcutaneous H1975 cell xenograft tumors. Volumes of tumors from vehicle control (●), BEZ235 (□), BIBW2992 (△) and BEZ235 + BIBW2992 (▼) groups (n = 5/group) were measured twice a week. Data are presented as means ± SEM (*, *P* < 0.05; **, *P* < 0.01; ***, *P* < 0.001; Student’s t-test)

Up to 50% of patients with acquired EGFR-TKI resistance possess a somatic T790 M mutation in the *EGFR* gene [9]. BIBW2992 (afatinib), a second generation, irreversible EGFR-TKI developed against HER2, HER4 and EGFR, including the T790 M mutation [5], has been approved by the FDA for the treatment of locally advanced and/or metastatic NSCLC. To determine whether BEZ235 also sensitizes NSCLC cells expressing the EGFR-T790 M mutant to BIBW2992, we assessed the viability and molecular profiles of H1975 cells simultaneously treated with BIBW2992 and BEZ235. Combined treatment with BIBW2992 and BEZ235 exerted synergistic cytotoxic effects compared with either single drug (Fig. 6a and b and Additional file 1: Figure S7a). Moreover, BIBW2992 blocked the phosphorylation of EGFR, AKT and ERK1/2, and slightly reduced the phosphorylation of p70S6K and 4EBP1, whereas BEZ235 potently suppressed the phosphorylation of p70S6K and 4EBP1 (Additional file 1: Figure S7b). Co-treatment with BEZ235 enhanced BIBW2992-induced cleavage of PARP and pro-caspase 3. Cyclin D was also decreased by both of BEZ235 and BIBW2992 (Fig. 6c). Similar results were obtained in CL97 cells, another NSCLC cell line expressing the EGFR-T790 M mutant (Additional file 1: Figure S7c).

We next investigated the in vivo antitumor efficacy of combined treatment with BEZ235 and BIBW2992 in xenograft mice. Nude mice were subcutaneously inoculated in the right flank with H1975 cells, and after tumor volumes reached ~ 50 mm^3^, were treated daily with vehicle control, 25 mg/kg of BEZ235, 5 mg/kg of BIBW2992, or both BEZ235 and BIBW2992 by oral gavage. Treatment with BEZ235 or BIBW2992 alone significantly reduced tumor volume compared with the control group, but co-treatment with BEZ235 and BIBW2992 exerted greater antitumor efficacy (Fig. 6d). Collectively, these results indicate that the dual PI3K/mTOR inhibitor, BEZ235, sensitizes EGFR-T790 M–expressing NSCLCs with acquired resistance to the irreversible TKI, BIBW2992.

## Discussion

**Fig. 7:**
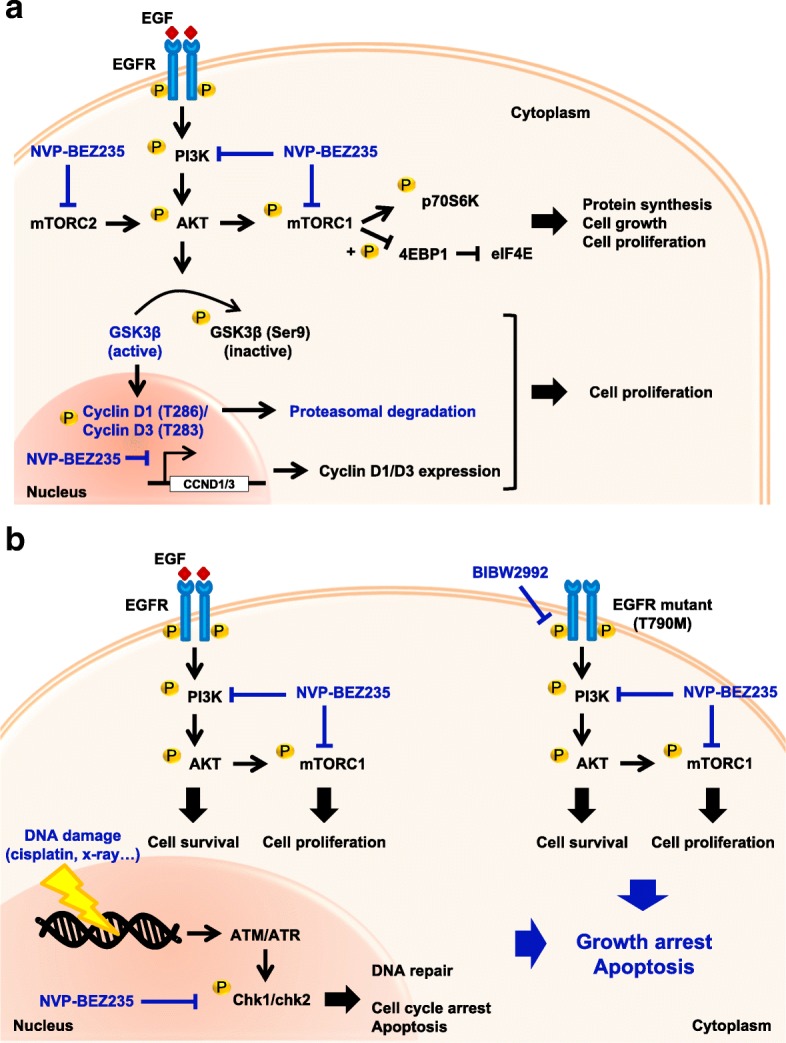
Models of BEZ235 effects in NSCLC. **a** A schematic representation showing that NVP-BEZ235 inhibits both PI3K and mTOR signaling induced by RTK, resulting in cell-cycle arrest at G1 phase through transcriptional and proteosome-mediated downregulation of cyclin D. **b** A schematic representation showing that the combination of NVP-BEZ235 and the DNA-damaging chemotherapeutic agent cisplatin is an effective antitumor strategy for NSCLC patients, and that the combination of NVP-BEZ235 with the EGFR-targeted therapeutic BIBW2992 is effective against NSCLC tumors with acquired resistance to TKIs caused by expression of the EGFR-T790 M mutant

Lung cancer is a highly heterogeneous disease and the leading cause of cancer death in the world [1–3]. Our results suggest that BEZ235, an oral, dual PI3K/mTOR inhibitor, offers a new avenue for the therapeutics of lung cancer. We found that BEZ235 suppressed cancer cell proliferation and the growth of lung cancer tumors regardless of their EGFR status by downregulating cyclin D members through both transcriptional inhibition and proteasome-mediated degradation (Fig. 7a). Combined with platinum-based chemotherapy, BEZ235 dramatically potentiated NSCLC cell death by suppressing Chk1 and Chk2, enhancing or prolonging DNA damage, and inducing apoptosis. In addition, BEZ235 combined with the EGFR-targeted therapeutic, BIBW2992, synergistically induced apoptosis of NSCLC cells with acquired resistance to TKIs caused by expression of the EGFR-T790 M mutant (Fig. 7b).

The activity of the PI3K/AKT/mTOR axis contributes to tumorigenesis, tumor progression, and resistance to therapy in most human cancer types [11–13, 27–29]. Using two independent in silico analyses, gene set enrichment analysis (GSEA) and a connectivity map (C-MAP), Ebi et al. showed that deregulation of mTOR signaling is associated with poor prognosis in lung adenocarcinoma [30]. Moreover, an analysis of gene expression profiles of NSCLC tumor samples and cell lines by Spoerke et al. revealed PI3K signaling alterations in NSCLC [31]. Notably, EGFR T790 M mutations and activation of alternative RTKs—major mechanisms for EGFR-TKI resistance—also ultimately activate PI3K/Akt/mTOR signaling [7, 10]. Thus, inhibition of the PI3K/Akt/mTOR pathway is predicted to exert antitumor activity in lung cancer. Indeed, our data support the conclusion that inhibition of Akt and mTOR by BEZ235 prevented mTOR-induced Akt activation and induced a striking antiproliferative effect in a broad range of NSCLC cells, regardless of their EGFR status. Unlike induction of apoptosis in primary effusion lymphomas with constitutively activated PI3K/Akt/mTOR signaling [32], gliomas [33] or HER2-amplified and PIK3CA-mutant breast cancer cells [16], BEZ235 monotherapy mainly induced cell-cycle arrest at G1 phase in NSCLC cells, even in CL141 cells that had lost PTEN expression. After withdrawing BEZ235, NSCLC cells re-grew. Moreover, BEZ235 may induce autophagic cell death in some lung cancer cell lines (e.g., A549 cells). Similarly, several reports have shown that dual PI3K/mTOR inhibitors used as monotherapies mainly lead to cytostatic antitumor effects in lung cancer and other cancers [17, 34], such as pancreatic cancer. Although BEZ235 alone has low cytotoxicity, the potent suppressive effect of BEZ235 on cell proliferation is still beneficial in controlling tumor growth in NSCLC.

In our study, we found that BEZ235 downregulated the expression of cyclin D1 and cyclin D3 in NSCLC cells via both transcriptional repression and proteasome-mediated degradation, resulting in cell cycle arrest at G1 phase. A transcription factor prediction analysis showed the presence of forkhead homeobox type O (FOXO) factor-binding sites in the promoter regions of D-type cyclins, and chromatin immunoprecipitation assays revealed that FOXO1 binds to the cyclin D1 promoter [35]. Notably, FOXO forkhead transcription factors are important downstream targets of PI3K/Akt signaling [36]. Moreover, it has been reported that repression of cyclin D1/D2 appears to be a general mechanism of growth suppression by the FOXO factors, FOXO1, FOXO3, and FOXO4 [35, 37]. Accordingly, the observed reduction in the mRNA expression of D-type cyclins may be mediated by FOXO factors through BEZ235-induced inhibition of PI3K/Akt activity. It has also been reported that GSK3β, another downstream target of PI3K/Akt signaling, is involved in the degradation of cyclin D protein [23, 38]. GSK3β phosphorylates cyclin D1 on Thr286 and cyclin D3 on Thr283, thereby targeting these proteins for proteosome-mediated degradation. In addition, Akt phosphorylates GSK3β on Ser9 and inactivates it [24]. Because BEZ235 blocks PI3K/Akt activity, it prevents GSK3β inactivation by Akt, resulting in cyclin D degradation. mTORC1 signaling has also been shown to regulate cyclin D1 translation by 4EBP1 [39]. Thus, inhibition of mTOR signaling by BEZ235 may be involved in the BEZ235-induced decrease in cyclin D expression as well. Collectively, these observations indicate that BEZ235 suppresses both PI3K/Akt and mTOR signaling, initiating multiple processes that converge on cyclin D downregulation and lead to growth inhibition.

Although BEZ235 alone was not sufficient to induce cell death in NSCLC, our data indicate that it synergistically increased cisplatin-induced apoptotic cell death and levels of the DNA-damage indicator γ-H2A.X, and reduced the activation of Chk1 and Chk2. Cisplatin, a prominent chemotherapeutic drug used to treat lung cancer, causes DNA damage in cancer cells by crosslinking purine bases in DNA to form intra-strand DNA adducts, and subsequently induces apoptosis. Nucleotide excision repair (NER) is responsible for removing cisplatin-induced DNA adducts [40]. In this process, single-strand DNA damage activates ATM (ataxia-telangiectasia mutated)-Chk2 and ATR (ataxia-telangiectasia Rad3-related)-Chk1 DNA damage sensor pathways, then recruits BRCA1 to DNA repair foci, and forms an NER complex that remove the adducts. If this process fails to repair the DNA damage, it triggers cell apoptosis [26, 41]. Although there is no evidence for a role for mTOR in NER, it has been reported that BEZ235 and other mTOR inhibitors suppress DNA double-strand break repair processes, including homologous recombination and non-homologous end joining, by inhibiting the catalytic subunit of DNA-dependent protein kinase (DNA-PKcs) and ATM, or through impaired recruitment of BRCA1 and Rad51 to DNA repair foci [42, 43]. NVP-BEZ235 has also been found to inhibit ATR [44]. Accordingly, BEZ235 may suppress NER-mediated repair of cisplatin-induced DNA adducts by inhibiting ATR activation and BRCA1 recruitment. Accumulating evidence supports the conclusion that BEZ235 is capable of sensitizing cancer cells to radiation, which causes double-strand DNA breaks [17, 42, 45]. Therefore, BEZ235 may be an effective sensitizing agent for lung cancer therapeutic strategies that induce DNA damage. It should also be noted in this context that overexpression of the PI3K/Akt pathway is one of the major mechanisms of cisplatin resistance [40], and cyclin D1 levels, which are suppressed by BEZ235, are inversely related to cisplatin sensitivity [46–48]. Thus, in addition to increasing DNA damage-induced apoptosis, combined treatment of NSCLC with cisplatin and BEZ235 may also prevent the occurrence of cisplatin resistance.

BEZ235 combined with the EGFR-targeting therapeutic, BIBW2992, also synergistically induced apoptosis in EGFR-T790 M mutant-expressing NSCLCs with acquired resistant to TKIs. BIBW2992 (afatinib) is a second-generation EGFR-TKI that irreversibly blocks both wild-type and mutant forms of EGFR through formation of covalent bonds with Cys797 in the pocket of the catalytic site [5]. PI3K/Akt/mTOR signaling is important in cell survival and is involved in the resistance to many cancer therapies, including RTK inhibitors [14]. It has been reported that the pro-apoptotic BCL-2 family member BIM is responsible for EGFR-TKI–induced apoptosis in lung cancers harboring oncogenic EGFR mutations [49]. Moreover, BEZ235 has been shown to increase BIM expression in various cancers by acting through inhibition of PI3K/Akt activity to induce FOXO3a activity [34, 50, 51]. Targeting the EGFR-T790 M mutant and the PI3K/AKT/mTOR pathway are both strategies for reversing EGFR-TKI resistance [52, 53]. In our study, we demonstrate that BEZ235 dramatically enhanced EGFR-TKI–induced apoptosis in EGFR-dependent NSCLCs, even those expressing the EGFR-T790 M mutant.

## Conclusions

Our data show that the dual PI3K/mTOR inhibitor, BEZ235, is an effective antitumor agent for treating lung cancer in vitro and in vivo, regardless of EGFR status. BEZ235 alone caused cytostatic antitumor effects, and when combined with DNA damage-based chemotherapy, sensitized lung cancer cells to the DNA-damaging effects of these agents. In addition, BEZ235 enhanced the effect of the EGFR-targeting therapeutic BIBW2992 on lung cancer cells with acquired resistant to TKIs caused by expression of the EGFR-T790 M mutant. Collectively, our findings suggest the future possibility of using BEZ235 as part of an anticancer strategy against lung cancer.

## Additional file

Additional file 1: **Figure S1.** The dose determination for BEZ235 and cisplatin in the in vivo study. **Figure S2.** Viability of BEZ235-treated NSCLC cell lines expressing wild-type EGFR or activating mutants of EGFR. **Figure S3.** Regrowth of BEZ235-treated NSCLC cells after withdrawal of the drug. **Figure S4.** Effects of BEZ235 on apoptosis, autophagy and cell cycle. **Figure S5.** β-catenin is not involved in the BEZ235-induced decrease in cyclin D. **Figure S6.** BEZ235 synergistically enhances cisplatin-induced apoptosis in NSCLC cells. **Figure S7.** BEZ235 synergistically enhances BIBW2992-induced apoptosis in EGFR-TKI–resistant NSCLC cells. (PDF 1190 kb)

## Data Availability

All data generated or analyzed during this study are included in this published article (and its additional file).

## Abbreviations

4EBP1: Eukaryotic translation initiation factor 4E binding protein 1
ATM: Ataxia-telangiectasia mutated
ATR: Ataxia-telangiectasia Rad3-related
EGFR: Epidermal growth factor receptor
EMT: Epithelial-mesenchymal transition
ERK1/2: Extracellular signal-regulated kinase 1/2
MAPK: Mitogen-activated protein kinase
mTOR: Mechanistic target of rapamycin
NER: Nucleotide excision repair
NSCLC: Non-small-cell lung cancer
p70S6K: Ribosomal protein S6 kinase
PARP: Poly (ADP-ribose) polymerase
PI3K: Phosphoinositide 3-kinase
RTKs: Receptor tyrosine kinases
STAT3: Signal transducer and activator of transcription 3
TKIs: Tyrosine kinase inhibitors
