# Supplementary material for: The dual PI3K/mTOR inhibitor BEZ235 restricts the growth of lung cancer tumors regardless of EGFR status, as a potent accompanist in combined therapeutic regimens
Source: J Exp Clin Cancer Res. 2019 Jul 1;38:282. doi: 10.1186/s13046-019-1282-0 (PMC6604380; doi:10.1186/s13046-019-1282-0)
Supplement: Supplementary file 1 — Figure S1. The dose determination for BEZ235 and cisplatin in the in vivo study. Figure S2. Viability of BEZ235-treated NSCLC cell lines expressing wild-type EGFR or activating mutants of EGFR. Figure S3. Regrowth of BEZ235-treated NSCLC cells after withdrawal of the drug. Figure S4. Effects of BEZ235 on apoptosis, autophagy and cell cycle. Figure S5. β-catenin is not involved in the BEZ235-induced decrease in cyclin D. Figure S6. BEZ235 synergistically enhances cisplatin-induced apoptosis in NSCLC cells. Figure S7. BEZ235 synergistically enhances BIBW2992-induced apoptosis in EGFR-TKI–resistant NSCLC cells. (PDF 1190 kb) [file 13046_2019_1282_MOESM1_ESM.pdf]

The dual PI3K/mTOR inhibitor BEZ235 restricts the growth of lung cancer tumors regardless of EGFR status, as a potent accompanist in combined therapeutic regimens

Yi-Ying Wu, Hung-Chang Wu, Jia-En Wu, Kuo-Yen Huang, Shuenn-Chen Yang, Si-Xuan Chen, Chao-Jung Tsao, Keng-Fu Hsu, Yuh-Ling Chen and Tse-Ming Hong

ONLINE DATA SUPPLEMENT

Figure S1.

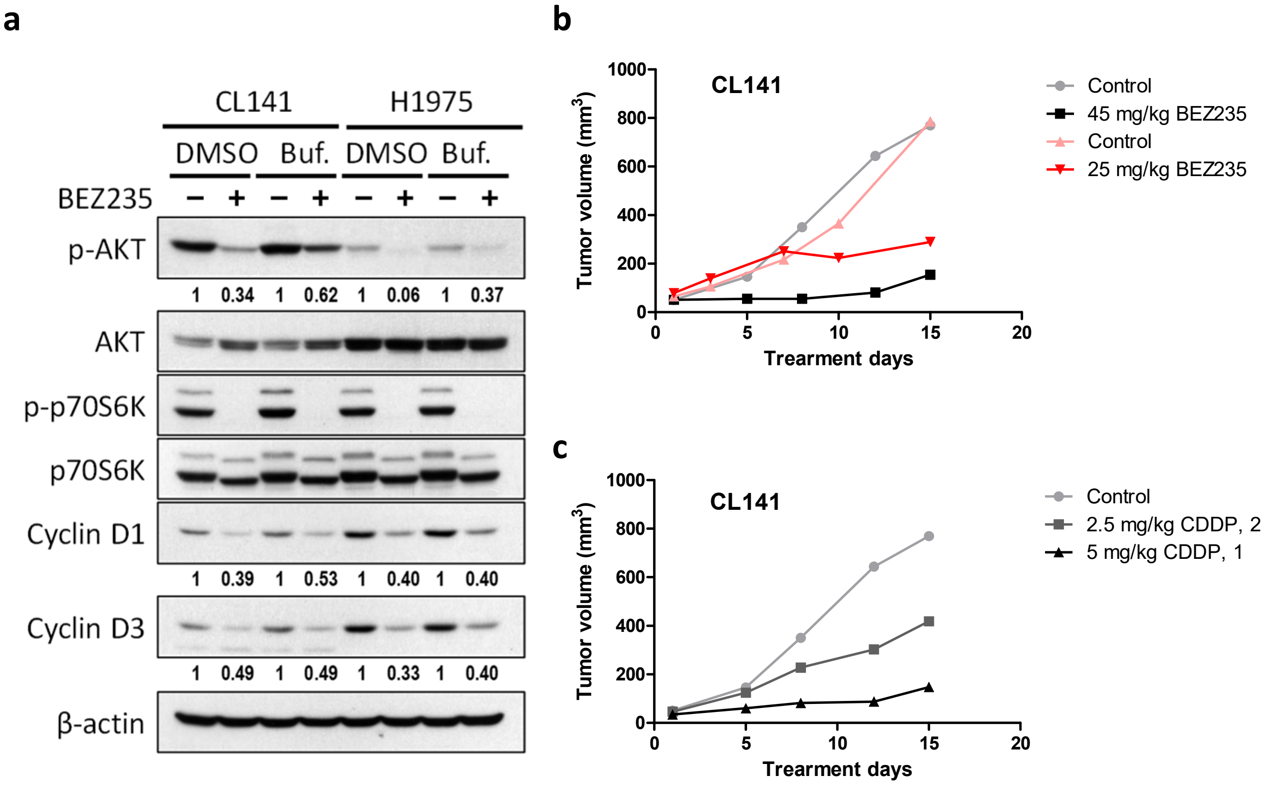

Figure S1. The dose determination for BEZ235 and cisplatin in the in vivo study. **a** Comparison of the activity of BEZ235 diluted in DMSO or the buffer with 0.5% methylcellulose and 0.1% Tween-80 *in vitro*. CL141 and H1975 cells were treated with 100 nM BEZ235 dissolved in DMSO or the buffer with 0.5% methylcellulose and 0.1% Tween-80 for 6 hours. Cell lysates were analyzed by Western blotting using the indicated antibodies. **b** BEZ235 (25 mg/kg or 45 mg/kg) was orally administered daily in mice bearing a subcutaneous CL141 cell xenograft tumor. N = 1 mouse per group. 25 mg/kg of BEZ235 was enough to suppress tumor growth and this dose also suited for combined treatment experiments **c** 2.5 mg/kg of cisplatin (CDDP) was intraperitoneally injected twice per week or 5 mg/kg of CDDP was injected once per week in mice bearing a subcutaneous CL141 xenograft tumor. N = 1 mouse per group. 2.5 mg/kg CDDP suppressed half of CL141 tumor growth while 5 mg/kg CDDP almost totally suppressed it. Then the condition, 2.5 mg/kg CDDP twice per week, was used for the combined treatment experiment.

Figure S2.

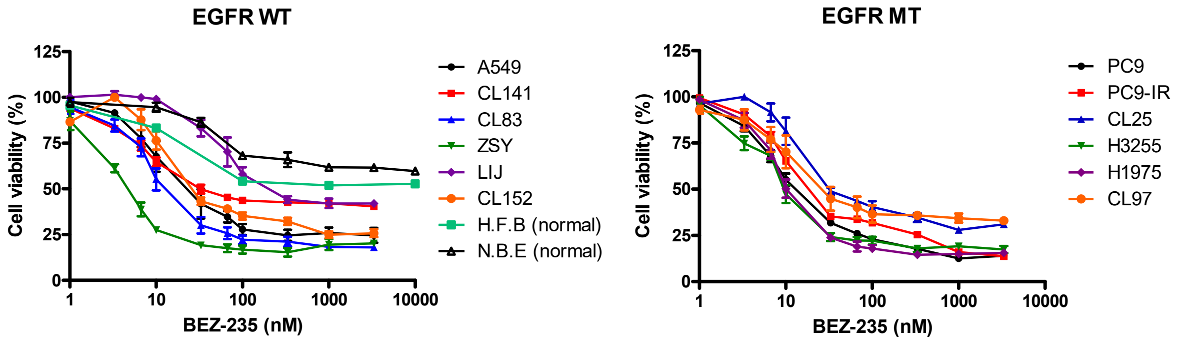

Figure S2. Viability of BEZ235-treated NSCLC cell lines expressing wild-type EGFR or activating mutants of EGFR. The viability of NSCLC cells treated with different concentrations of BEZ235 for 72 hours was assessed using the WST-1 assay. H.B.F, human normal bronchial fibroblasts; N.B.E, human normal bronchial epithelia. Values are reported as means ± SD.

Figure S3.

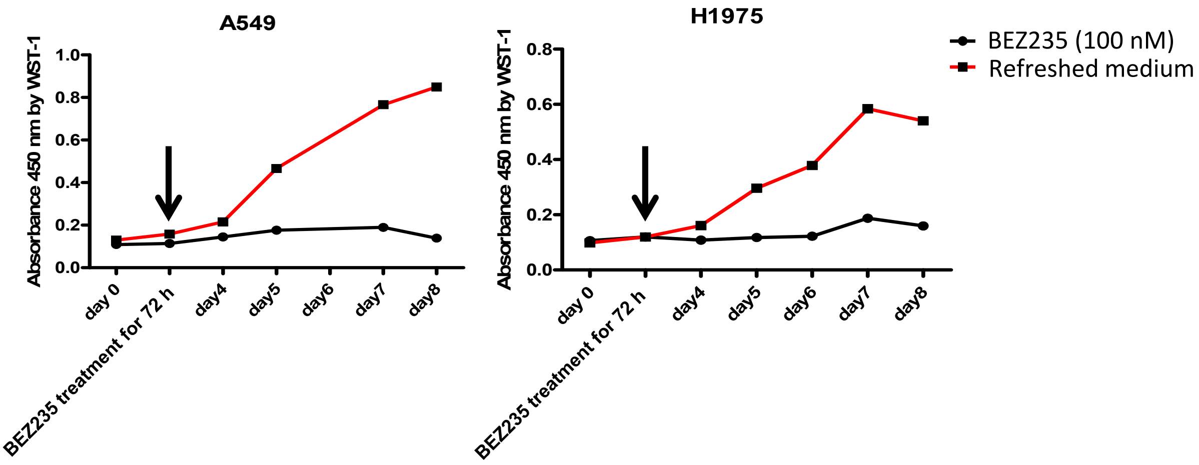

Figure S3. Regrowth of BEZ235-treated NSCLC cells after withdrawal of the drug. After treating with BEZ235 for 72 hours, A549 and H1975 cells were cultured in fresh complete media without BEZ235 for an additional 5 days. Live cells were detected daily by WST-1 assay.

Figure S4.

a

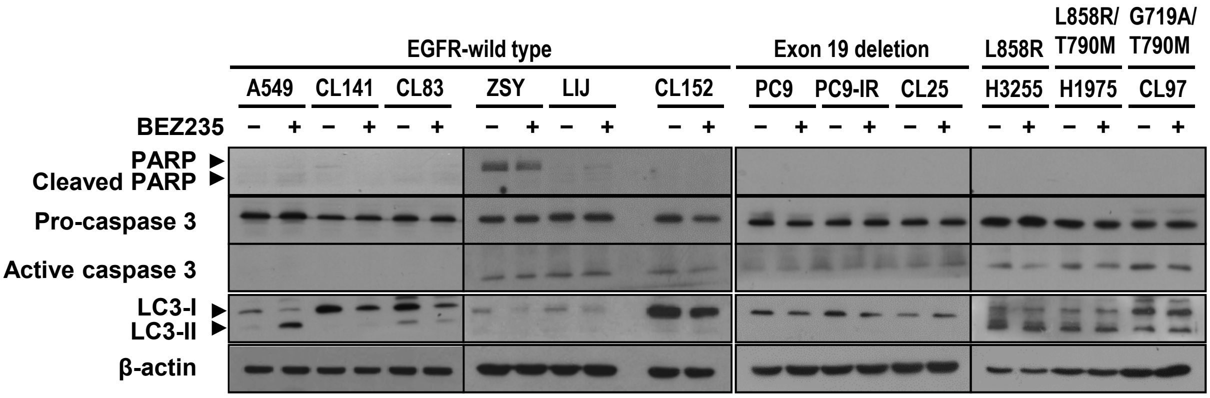

b

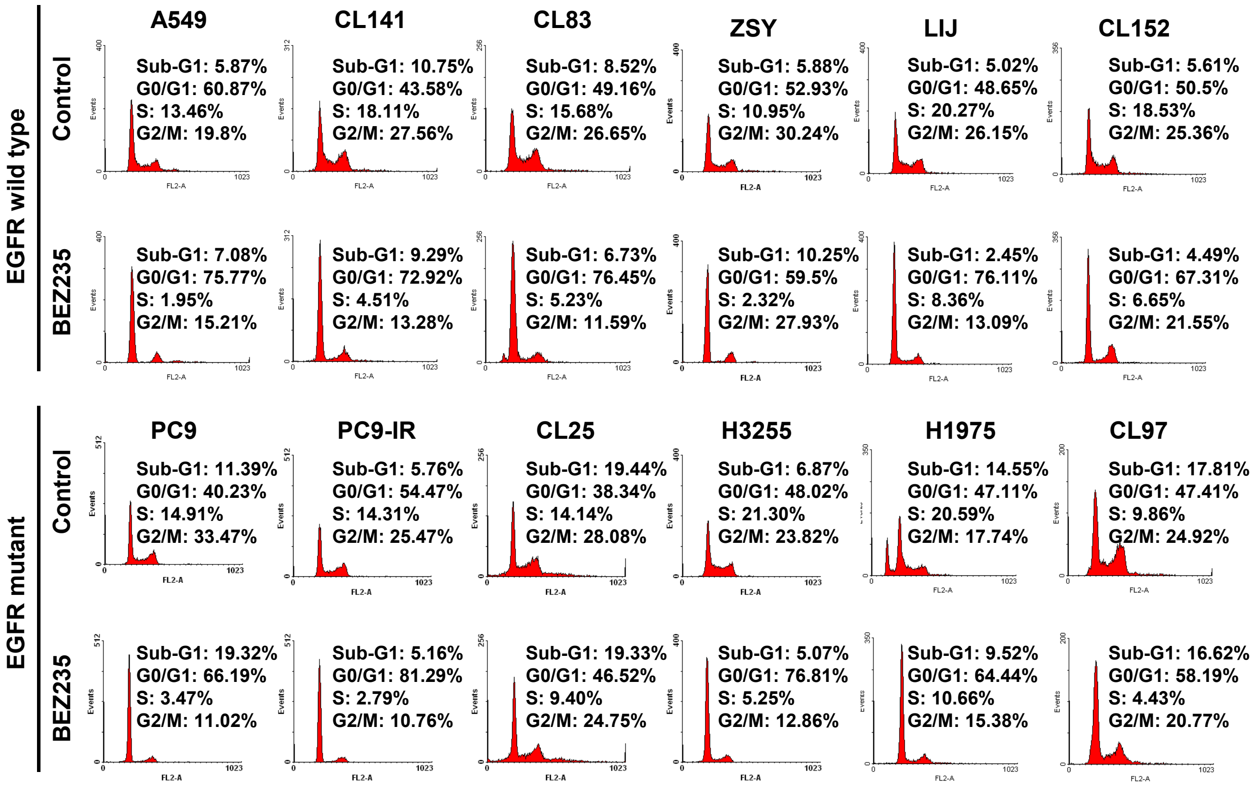

Figure S4. Effects of BEZ235 on apoptosis, autophagy and cell cycle. **a** Effects of BEZ235 on apoptosis and autophagy. Cells were treated with 100 nM BEZ235 for 24 hours. The apoptotic indicators, cleaved PARP and caspase 3, and the autophagic marker LC3 were detected using the indicated antibodies. **b** Cell-cycle analyses of BEZ235-treated NSCLC cells. NSCLC cells were treated with 333 nM BEZ235 for 24 hours, and then the cell-cycle distribution was analyzed by flow cytometry. The percentage of cells in each phase of the cell cycle is shown.

Figure S5.

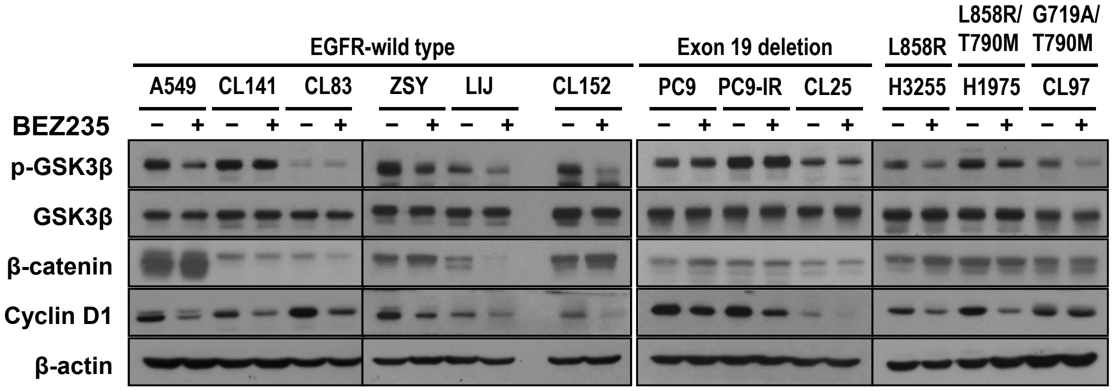

Figure S5. β-catenin is not involved in the BEZ235-induced decrease in cyclin D. NSCLC cells were treated with 100 nM BEZ235 for 6 hours. Cell lysates were analyzed by Western blotting using the indicated antibodies. β-actin was used as an internal control.

Figure S6.

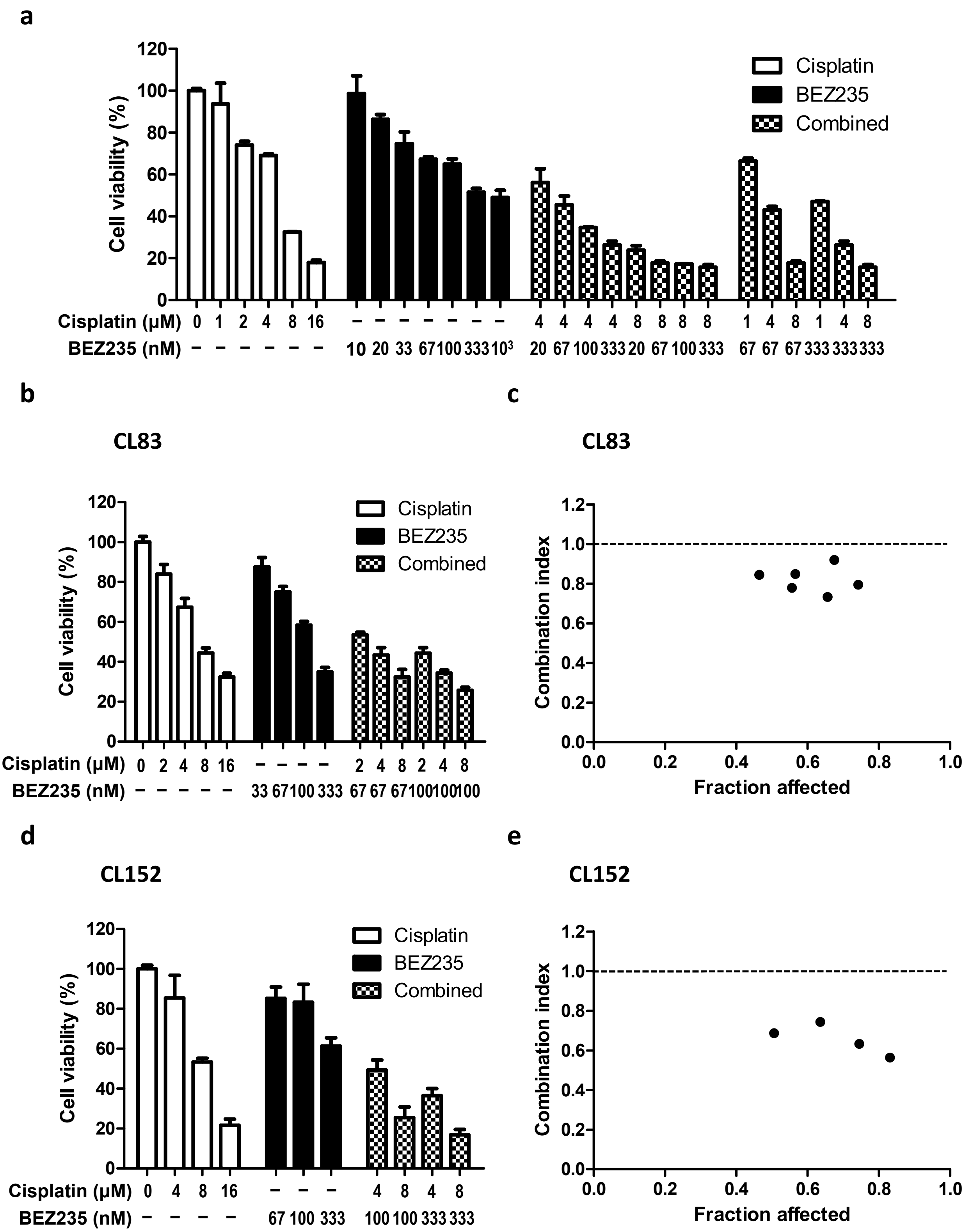
a
Cell viability (%)
Cisplatin
BEZ235
Combined
Cisplatin (μM)
BEZ235 (nM)
b
CL83
c
CL83
Combination index
Fraction affected
d
CL152
e
CL152

Figure S6. (continued)

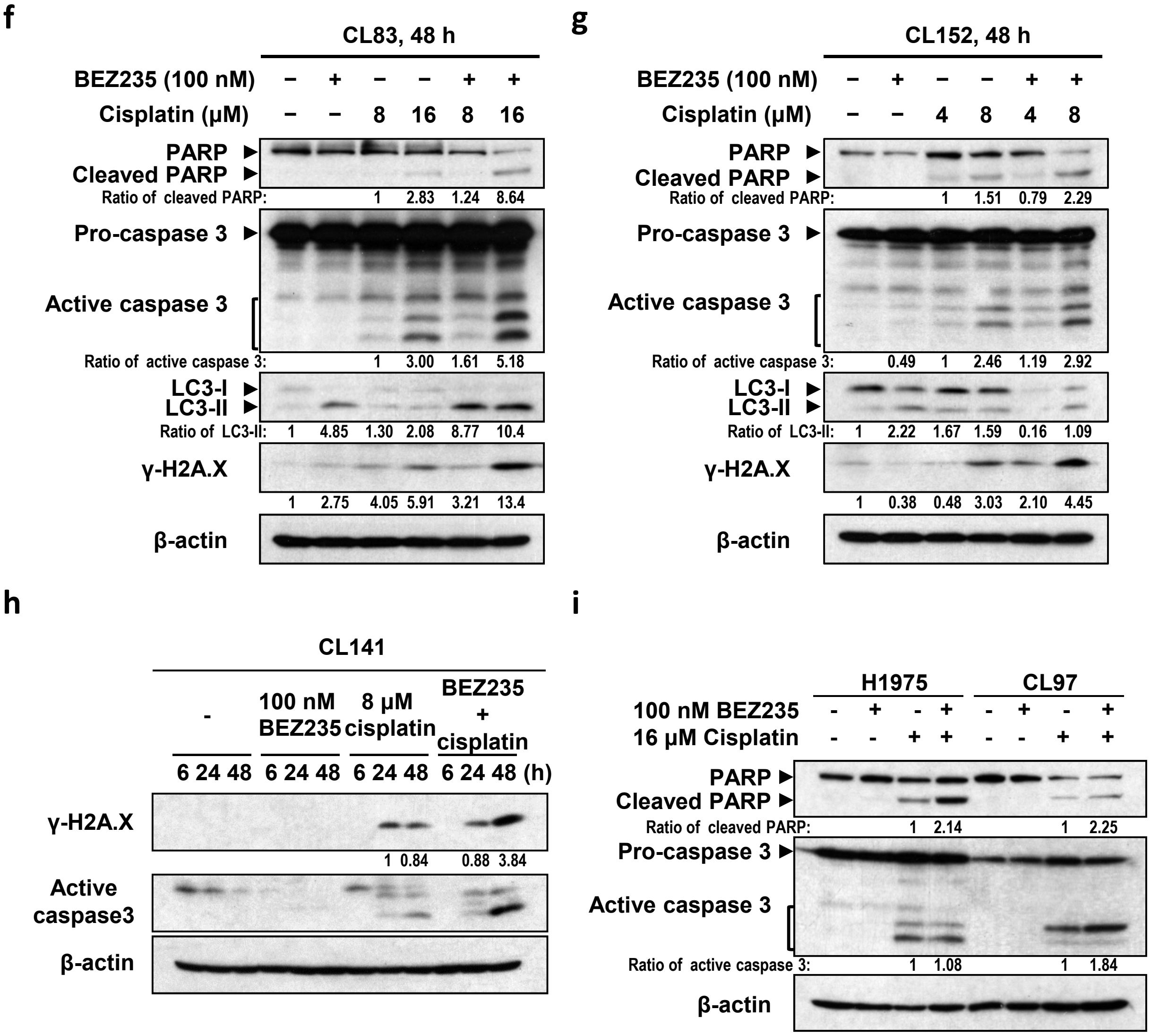

Figure S6. BEZ235 synergistically enhances cisplatin-induced apoptosis in NSCLC cells. **a** CL141 cells were treated with the indicated concentrations of cisplatin and BEZ235, either alone or together, for 48 hours. Viability was analyzed using the WST-1 assay. The results of combined treatment were used to calculate the combination index (CI) and the CI values were shown in Fig. 5b. CL83 (**b**, **c**, **f**) and CL152 (**d**, **e**, **g**) cells were treated with the indicated concentrations of cisplatin and BEZ235, either alone or together, for 48 hours. Viability was analyzed using the WST-1 assay (**b**, **d**). The combination index was calculated as described in Materials and Methods (**c**, **e**). Levels of cleaved PARP, active caspase 3, LC3, and γ-H2A.X in cell lysates were analyzed by Western blotting (**f**, **g**). **h** CL141 cells were treated with 8 μM cisplatin and 100 nM BEZ235, either alone or together, for the indicated time. Levels of active caspase 3, γ-H2A.X, and β-actin in cell lysates were analyzed by Western blotting using the indicated antibodies. **i** H1975 and CL97 cells were treated with the indicated concentrations of cisplatin and BEZ235, either alone or together, for 48 hours. Levels of cleaved PARP, active caspase 3, and β-actin in cell lysates were analyzed by Western blotting. The related expression levels of protein, quantified by ImageJ as described in Materials and Methods, were shown below their corresponding blots.

Figure S7.

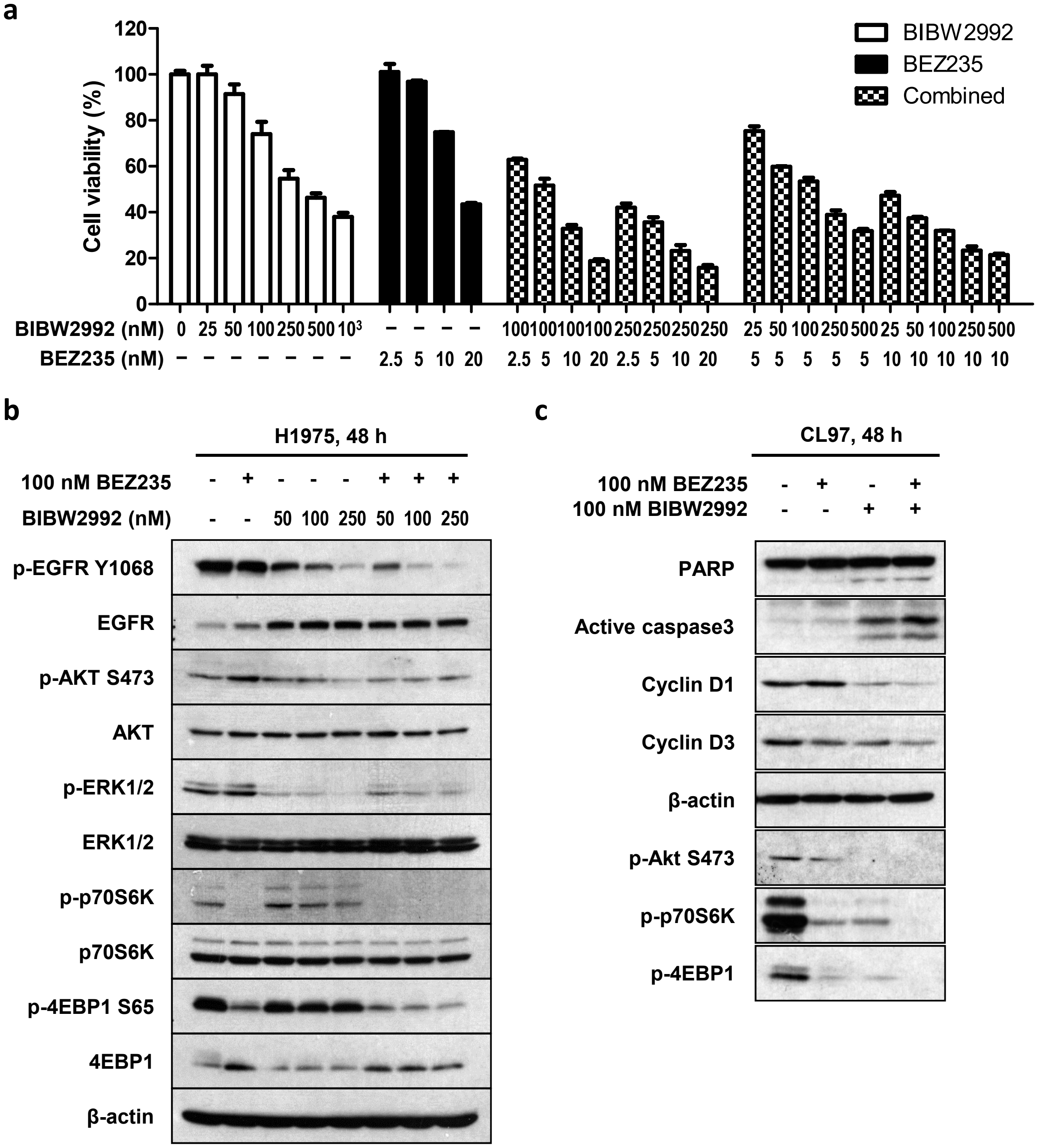

Figure S7. BEZ235 synergistically enhances BIBW2992-induced apoptosis in EGFR-TKI–resistant NSCLC cells. **a** H1975 cells were treated with the indicated concentrations of BIBW2992 and BEZ235, either alone or together, for 72 hours. Viability was analyzed using the WST-1 assay. The results of combined treatment were used to calculate the CI values, shown in Fig. 6b. **b** H1975 cells were treated as described in Figure 6c. Levels of the indicated proteins in cell lysates were analyzed by Western blotting using the indicated antibodies. **c** CL97 cells were treated with 100 nM BIBW2992 and BEZ235, either alone or together, for 48 hours. Levels of cleaved PARP, active caspase 3, cyclin D1/D3, phosphorylated Akt, phosphorylated p70S6K, phosphorylated 4EBP1, and β-actin in cell lysates were analyzed by Western blotting using the indicated antibodies.
